# Editorial Highlights from the Comorbidities and Complications of Cerebral Palsy Special Issue

**DOI:** 10.3390/jcm12165329

**Published:** 2023-08-16

**Authors:** Monica S Cooper, Christine Imms

**Affiliations:** 1 Department of Neurodevelopment & Disability, Royal Children’s Hospital, 50 Flemington Road, Melbourne, VIC 3052, Australia; 2 Neurodisability and Rehabilitation, Clinical Sciences, Murdoch Children’s Research Institute, 50 Flemington Road, Melbourne, VIC 3052, Australia; 3 Department of Paediatrics, University of Melbourne, 50 Flemington Road, Melbourne, VIC 3052, Australia

Cerebral palsy is a life-long condition and the most common cause of physical disability in childhood. Despite evidence of falling prevalence in some Western countries, cerebral palsy remains a global concern. Cerebral palsy is a clinical description, rather than a discrete diagnosis, and the aim of our Special Issue was to provide readers with advances in our understanding of associated comorbidities and complications. We collated research from different disciplines that addressed issues through different stages of life. In this editorial, we will draw the readers’ attention to several of the papers to highlight specific life-stage issues, along with the need to address global inequities and, importantly, the long-term health consequences of growing up and living with cerebral palsy. 

## 1. The Early Years

Starting with infants at high risk of cerebral palsy, a study of parents and their experiences with their infants found that most mothers and fathers, but not all, were able to be engaged with their infants [1]. Given the crucial nature of parent–infant interactions to child development and family wellbeing, this information highlights the need to situate our roles and interventions within a family-centred context and to provide interventions that support early and ongoing parent–child interactions. Additionally, in this early life stage, a lack of evidence-based interventions for infants with communication impairments led Ward et al. (2022) to try a multi-modal intervention with pre-linguistic infants with CP (starting at 16 months), with encouraging results in their targeted areas of language and speech production [2].

A prospective, longitudinal study on medical gastrocnemius morphology and growth showed slower rates of muscle growth, especially in the first two years of life in children with cerebral palsy functioning within the Gross Motor Function Classification System (GMFCS) level II-III, compared to those functioning within GMFCS level I. These results highlight that reduced muscle growth occurs early and that goals and treatment planning should also be considered early [3].

## 2. Cells, Biology and Pathophysiology

With the rising interest in stem cell infusions, a scoping review of the outcome measures used post-stem cell infusions showed that most measures did not have the requisite measurement properties to validly measure a change following the intervention. The authors’ recommendations about the outcome measure selection include ensuring that the captured outcomes aligned with the priorities of the cerebral palsy community, such as quality of life [4]. Another paper focused on biomarkers and the role of inflammation in cerebral palsy, which are being increasingly studied. This paper provides supporting evidence suggestive that inflammation may persist into the adult years, likely contributing to the pathogenesis of cerebral palsy and associated complications [5].

A review of the pathophysiology of acquired hip dysplasia in cerebral palsy provided considerations for determining the best timing and types of paediatric surgical interventions, as well as broader reflections on the hip surveillance program [6]. For the surgical correction of scoliosis, experiences of a peri-operative pathway to guide families through shared decision-making and the optimisation of post-operative care were highlighted in a publication of a multifaceted service model aimed to improve patient and caregiver experiences as well as improved outcomes [7].

## 3. Life Course Issues

Cerebral palsy registers continue to provide valuable information about longitudinal reporting. Data from the first population-based cerebral palsy register in Bangladesh were published. These results highlight the stark disparities in accessing education and rehabilitation services, which then impact negatively on participation and functional outcomes [8].

The unmet needs of adults with cerebral palsy were highlighted in three papers, with a reminder that we need a life-course approach to service provision [9]. Significant changes in both what health care is and how it is provided are also needed to manage the high prevalence of anxiety and depression in adults with cerebral palsy [10]. Lastly, to better delineate mortality causes in cerebral palsy, recommendations were made to detail the mechanisms of mortality in addition to the underlying associated cerebral palsy. This way, we can better understand the complications that evolve and lead to preventable early death in individuals with cerebral palsy [11].

Although we now have decades of cerebral-palsy-specific research, we still need to deepen our knowledge about the broad spectrum of impacts of the condition. We also do not yet have a suite of effective interventions that address the range of body system functions or community participation needs. There is still much work to be done.
